# A spatially supported forced-choice recognition test reveals children’s long-term memory for newly learned word forms

**DOI:** 10.3389/fpsyg.2014.00164

**Published:** 2014-03-06

**Authors:** Katherine R. Gordon, Karla K. McGregor

**Affiliations:** DeLTA Center, Department of Communication Sciences and Disorders, University of Iowa, Iowa CityIA, USA

**Keywords:** word learning, fast mapping, memory, word form, recognition

## Abstract

Children’s memories for the link between a newly trained word and its referent have been the focus of extensive past research. However, memory for the word form itself is rarely assessed among preschool-age children. When it is, children are typically asked to verbally recall the forms, and they generally perform at floor on such tests. To better measure children’s memory for word forms, we aimed to design a more sensitive test that required recognition rather than recall, provided spatial cues to off-set the phonological memory demands of the test, and allowed pointing rather than verbal responses. We taught 12 novel word-referent pairs via ostensive naming to sixteen 4- to 6-year-olds and measured their memory for the word forms after a week-long retention interval using the new spatially supported form recognition test. We also measured their memory for the word-referent links and the generalization of the links to untrained referents with commonly used recognition tests. Children demonstrated memory for word forms at above chance levels; however, their memory for forms was poorer than their memory for trained or generalized word-referent links. When in error, children were no more likely to select a foil that was a close neighbor to the target form than a maximally different foil. Additionally, they more often selected correct forms that were among the first six than the last six to be trained. Overall, these findings suggest that children are able to remember word forms after a limited number of ostensive exposures and a long-term delay. However, word forms remain more difficult to learn than word-referent links and there is an upper limit on the number of forms that can be learned within a given period of time.

## INTRODUCTION

Extensive research on word learning reveals that young children can successfully link a word to its referent after only a few exposures (Katz et al., 1974; Carey, 1978, 2010; Markman, 1989; Waxman and Booth, 2003; Gleitman et al., 2005; Dewar and Xu, 2007) and can generalize that link to untrained exemplars of the referent (Markman, 1989; Woodward et al., 1994; Booth and Waxman, 2002; Perry et al., 2009). These findings have contributed to the predominant characterization of children as expert word learners. However, the word-referent link is only one piece of the word-learning problem. Equally important is the ability to recognize and produce the word form correctly. While researchers have investigated the specificity of infants’ representations of word-forms (reviewed below), there is little literature exploring preschool-age children’s representations of forms. However, given the theoretical, as well as clinical importance of understanding how preschool-age children incorporate new words into their vocabularies, it is important to investigate word-form learning in this age group.

From birth, infants are able to discriminate between the meaningful phonetic contrasts of a wide variety of languages and they maintain the ability to discriminate phonetic contrasts that are meaningful in their native language(s) over the course of development (see Werker and Curtin, 2005 for a review). For our purposes, it is telling that the ability to discriminate between native speech sounds manifests itself differently during word learning tests than during simple phonetic discrimination tests. For example, Werker and Stager (2000) found that 14 month-old infants were able to discriminate between the word forms “bih” and “dih” when viewing a checkerboard pattern. However, when the infants were habituated to the word “bih” paired with a potential referent, they failed to discriminate “bih” from “dih”. Both Werker and Stager (2000) and Swingley and Aslin (2000) have suggested that this is because the task of matching a word to a referent places extra demands on the child and, thus, distracts from their ability to focus on the specific phonetic features of the word form (see Werker and Fennell, 2006 for a review) and, indeed, different tasks (Yoshida et al., 2009), higher word familiarity (Swingley and Aslin, 2000), and richer training (Rost and McMurray, 2009) yield better performance.

Additional research suggests that infants may have difficulty discriminating between similar words forms because they encode partial representations of forms after a limited number of exposures. Kay-Raining Bird and Chapman (1998) presented novel word-referent pairs to 13- to 16-month old infants and tested their willingness to associate the target object with variations of the target word. They found that infants accepted many variations of the target word as a label for the target object, but their willingness to link the word-form and object decreased as the word-form became more dissimilar from the target. This suggests that infants of this age are able to encode word-forms after a limited number of exposures, but they encode only partial representations of these forms. However, the ability to map words with greater phonetic specificity after a limited number of exposures improves over time. There is evidence that 20 month-olds do so (Werker and Tees, 1999; Werker et al., 2002) as well as 14 month-olds who have larger vocabularies (Werker et al., 2002). Werker and Tees (1999) posit that this occurs because children are learning more words that are phonetically similar to one another, so they must encode more exact representations of those forms to differentiate them (also see Metsala and Walley, 1998). Other researchers provide a different explanation for this shift. Namely, the more practiced a child is at producing various speech sounds, the better that child will be at encoding and reproducing novel words that are composed of those speech sounds (Keren-Portnoy et al., 2010).

Currently, we know little about whether children older than 2 years-of-age also encode partial representations of word-forms after a limited number of exposures. Given past findings with infants, it seems likely that older children, by virtue of their larger vocabularies, would be more likely to encode specific representations of word forms during initial learning. Therefore, they should readily reject phonetically similar forms (e.g., near neighbors) as labels for trained referents. However, with a few notable exceptions (reviewed below) this hypothesis has yet to be tested empirically. The lack of research on word-form learning among children is, in part, due to a lack of sensitive methodology to test children’s memory for the forms. While research with infants has included tests of whether they will accept variations of the target words as acceptable labels for trained objects, similar methods have not been widely implemented in research with children. Typically in word learning studies with children, they are familiarized with a series of novel words and their referents (and the referents are typically objects) and are then asked to demonstrate their knowledge of the link between these words and referents by selecting each referent from an array when it is named (e.g., Where is the blicket?). Such alternative forced choice (AFC) tests do assess children’s memory for word forms to some extent in that they must recognize the word form to be able to select the word referent. However, these tests are not very sensitive in that they do not reveal the specificity of that word form in memory.

When they are included, tests designed specifically to measure word-form learning and memory in children typically involve asking them to name newly trained referents when they are presented (e.g., What is this one called?). While these verbal recall tests do provide additional information about children’s memory for word forms, Swingley and Aslin (2000) identified significant limitations of these tests. Namely, the child’s correct pronunciation of the word form does not necessarily mean that she would not accept other pronunciations of the word form as labels for the target referent. Conversely, the child’s mispronunciation or failure to produce the word form does not necessarily mean that she would not be able to identify another person’s correct pronunciation of the word. Given these limitations, the methodologies typically used to assess children’s memory for word forms are not fully adequate.

A related issue is that children generally perform worse in recall tests that assess their memory of word forms than recognition tests that assess their memory for word-referent links (Dollaghan, 1985; Weismer and Hesketh, 1996; Gray, 2003, 2004; Gupta, 2005; Horst and Samuelson, 2008). However, it is not clear whether this performance gap reflects differences in the learnability of links (which initially requires a one-to-one association) and word forms (which are sequenced, fleeting perceptual targets) or whether the gap reflects differences in the demands of the tests (McMurray et al., 2012). In general, recall tests entail higher memory demands than recognition tests for both children and adults (Craik and McDowd, 1987); therefore, recall tests used to assess word learning might underestimate children’s knowledge of word forms. In fact, adding memory cues to these tests does improve children’s performance, suggesting that even if children are unable to produce the forms initially, they do have some memory of the forms (Capone and McGregor, 2005; Munro et al., 2012). In addition to different memory demands, recall and recognition tests place different demands on children’s motor skills. Typically, recognition tests require children to pick up or point to the target object, while recall tests require children to produce the word form (Dollaghan, 1985; Gray, 2004; Horst and Samuelson, 2008). Developmental research has provided evidence that manual motor skills develop earlier and are easier for most children to execute than the motor skills required to produce speech sounds (see Smith and Goffman, 2004), and children’s ability to produce all native speech sounds in an adult-like manner has a protracted development that extends well into middle childhood (Munson et al., 2011). Thus, while young children may be able to recall the correct word form, they may have difficulty producing it. Finally, an obvious difference between recall and recognition tests is that only the latter allows for correct responding via chance. Therefore, recognition tests of word-referent links and verbal recall tests of word forms are not comparable.

Given these past limitations on testing children’s memory for word forms, our primary goals for the current study were to determine whether children demonstrate memory for newly trained word forms under reduced task demands and whether memory for word forms is as robust as memory for word-referent links. To address our primary goals, we designed a three alternative forced choice (3AFC) test of word forms that reduces memory and motor demands on children and is more comparable to traditional tests of word-referent links. In this test, children see a newly trained referent and are queried about their memory for the word form that names it (i.e., Do you remember what this one is called?). However, instead of requiring children to recall the form, we present three word forms for children to choose from (i.e., Is it a dorb, a vorb, or a zinnip?). In designing this measure of children’s memory for word forms, our aim is not to replace past measures, as it is still useful to evaluate when children are able to recall and produce learned word forms. Instead our aim is to develop a test that is sensitive to the development of children’s memory for the forms before they are fully able to verbally recall them.

As we designed the new test, we thought carefully about the three alternatives that would be presented to children. In many AFC tests of word-referent links, children are presented with a variety of novel objects: one or more that were trained; one or more that they were exposed to during training, but were never named; and one or more that were never exposed (Dollaghan, 1985; Horst and Samuelson, 2008; Vlach and Sandhofer, 2011; Goldenberg and Sandhofer, 2013). The exposed/unnamed object(s) is typically included to ensure that children are not responding on the basis of familiarity alone, and the unexposed object(s) is included to reduce the likelihood that children will select the target based on chance. Given this precedent, we elected to present children with three alternatives in our test of word forms: a trained novel word form (the target word), a near neighbor novel word that varied from the target in either onset, medial, or final consonant only, and a maximally different novel word that differed from the target in both number of syllables and majority of segments. Similar to tests of word-referent links, the maximally different novel word is included to decrease the likelihood that children would select the target by chance. The near neighbor serves to reduce the chance that children are selecting the target based on a general familiarity with the word form (such as knowing the right number of syllables, or some of the phonemes of the word form). By including both the target word and a near neighbor in our test of word form, we can gain a better understanding of whether preschool age children are able to recognize the specific word form, or if, similar to infants, they only encode a partial representation of the form after a limited number of exposures.

While providing children with three options does make tests of word forms and word-referent links more comparable, the options presented to children in the word-form test are fleeting auditory stimuli as opposed to the stable visual stimuli commonly presented in tests of word-referent links. For the current test to be a valid assessment of children’s memory for word forms, children must have the phonological working memory capacity to maintain all three forms while they select one. Past evidence suggests that phonological working memory is composed of two components: the phonological store and the subvocal rehearsal process (see Baddeley et al., 1998). Phonological information enters the phonological store after it is perceived auditorily, but rehearsal is needed to maintain that information in working memory (Baddeley et al., 1975). While young children can maintain verbal information in the phonological store for a very limited amount of time, they do not show evidence of the subvocal rehearsal process until around age seven (Gathercole and Hitch, 1993). In fact, after they are presented with a list of familiar words, 3- and 4-year-olds are only able to repeat, on average, 2–3 items from the list (Chi, 1976; Hulme et al., 1984). Furthermore, the characteristics of the individual words can affect how many words children can recall. For example, as the number of syllables per word increases, the number of words children can remember decreases (Nicolson, 1981; Hulme et al., 1984; Hitch et al., 1988). Given that 2–3 familiar words is the average preschool-age children can recall, it is likely that some children would have difficulty maintaining three novel word forms in working memory during our 3AFC test of word forms, especially when those forms are multisyllabic.

A few researchers have developed innovative strategies for testing children’s memory for word forms that reduce demands on children’s phonological working memory. One strategy is to teach children novel word-referent links and then, across different trials, asked if various labels could be applied to a specific trained referent via yes/no questions (Weismer and Hesketh, 1996, 1998; Alt et al., 2004; Alt and Plante, 2006). For example, Alt and Plante (2006) presented the target label, a one-syllable foil that was phonologically related to the target, a two-syllable foil that was phonologically related to the target, and either a one- or two-syllable foil that was not phonologically related to the target across four different trials (e.g., Is this a fuvis?). In this test, children were not required to make verbal responses, but instead pressed a button to indicate whether the word form could be applied to the object or not. Typically developing children performed fairly well in this test and performed better than children of this age usually perform in traditional verbal recall test of word forms. This method is similar to the one that Kay-Raining Bird and Chapman (1998) used with infants in that they presented a different word-form during each trial and the infant could indicate (through looking at or touching the target object) whether they accepted each form as a label for the target object. While this method does reduce phonological working memory demands on children, it is time consuming in that children must complete a separate trial for each word form assessed. Also, given that children are not presented all options during the same trial, it is difficult to directly compare results obtained through this method to children’s performance in traditional AFC tests of word-referent links and generalization of those links.

A different solution appears in Nash and Donaldson (2005) in which they taught 4- to 9-year-old children (with and without language impairment) eight unfamiliar but real English word-referent links. They then tested children’s memory for word forms with both a traditional verbal recall test and an AFC recognition test. In the AFC recognition test, they showed children the target referent along with a paper containing a grid of boxes. The experimenter produced four minimally distinctive word forms for the child to choose from while pointing to one of the boxes for each word form they produced. Children were encouraged to place a sticker on the box that corresponded with the correct word form. This methodology is similar to one used by Storkel (2001) to test children’s memory for words when presented with semantically related (rather than phonologically related) forms. After exposing 3- to 6-year-olds to novel words and their referents in the context of a narrative, she offered three of the trained words (two semantically related words and an unrelated word) as alternative labels for a given referent, pointing to a yellow square as each word was presented. The child could indicate his memory for the word by touching the square that corresponded to his choice. The methods employed by Nash and Donaldson (2005) and Storkel (2001) reduce demands on phonological working memory, but also allow presentation of all options during the same trial. Additionally, both methods exploit space as a cue for grounding verbal memory, a notion consistent with recent work by Samuelson et al. (2011) in which space was used to ground initial encoding of new words. Furthermore, both methods allow children to respond manually rather than verbally.

**FIGURE 1:**
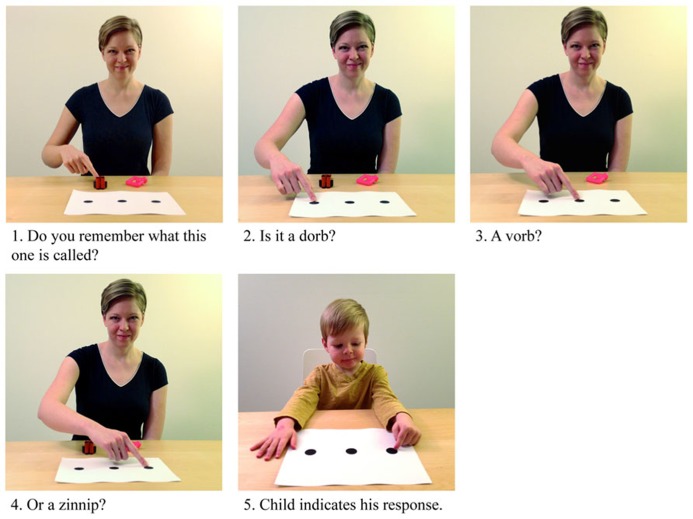
**Procedure used for the three alternative forced choice form test**.

Although Nash and Donaldson (2005) did not conduct a direct comparison of children’s performance in the verbal recall and recognition tests, visual inspection of the means and ranges suggests that children performed better on the recognition test across all testing sessions and conditions. However, despite these promising results, Nash and Donaldson (2005) found a wide range of performance. Additionally, even among those who were not affected by language impairment, there were children whose performance approached floor. Whereas this could be a reflection of true variation in word learning ability, it is possible that the test employed by Nash and Donaldson (2005) remained too demanding for the children’s limited phonological memory capacities. Given that children can often hold only 2 or 3 forms in phonological memory, we elected to present three rather than four alternatives in the task we designed (similar to Storkel, 2001). As these other researchers have done, we went further to off-set memory demands by adding visual-spatial supports to our word-form test. In our version, the researcher showed the child a piece of paper with three large black dots on it and pointed to one of the dots as she produced each word form. The child could then respond by pointing to the dot that corresponded to his or her answer, eliminating the need for a verbal response (see **Figure 1**).

Given what we hypothesized would be a more sensitive test of word-form knowledge, we aimed to “test the limits” by applying the test after a one-week retention interval. This is unlike previous research on word learning, which is largely limited to very short retention intervals. For example, children’s memory for word-referent links is typically assessed immediately after training and children tend to perform very well under these testing conditions (Golinkoff et al., 1992; Mervis and Bertrand, 1994; Spiegel and Halberda, 2011). However, recent research has provided evidence that retention of word-referent links is poor after a week and, in some cases, even after 5-min (Horst and Samuelson, 2008; Munro et al., 2012; Vlach and Sandhofer, 2012) and that a more extensive level of training or a stronger level of encoding is needed for long-term retention (Horst et al., 2006; McGregor et al., 2007). For example, the experimenter’s ostensive naming of the object referents (Horst and Samuelson, 2008; Jaswal and Markman, 2003) and the child’s manipulation of those objects (Yoshida and Smith, 2008) have been shown to improve long-term retention. As for word forms, children (Booth et al., 2008) and adults (McGregor et al., 2013) are typically at floor in traditional verbal recall versions of these tests even immediately after training; thus, longer-term retention intervals are rarely considered. In one of the few studies in which children’s long-term memory of word forms was tested, 8-year-olds were trained and tested on 20 novel word-referent links during three weekly sessions (McGregor et al., 2007) and were then tested one month after the third session. During each test, they were asked to name and define each referent. Children’s performance after the first session was very poor; they were only able to successfully name an average of 0.97 out of 20 referents. Their performance gradually improved until they were naming an average of 8.15 referents during the last session. However, at this point it is unknown if children require more extensive training of word forms than word-referent links to foster long-term retention or whether seemingly poorer short- and long-term retention of word forms is attributable to test methods that are not comparable to tests of word-referent links. To begin to resolve this question, the current study explores the retention of trained word-referent links and their generalization as well as retention of trained word forms over a 1-week interval using comparable test methods.

For the current study, we taught children 12 novel word-referent links via ostensive naming and measured their memory one week later through three 3AFC tests: a form test (which assessed memory for word forms), a link test (which assessed memory for word-referent links), and a link-generalization test (which assessed children’s willingness to apply trained forms to untrained exemplars). The form test used visual-spatial supports (i.e., the three dots) to minimize phonological memory demands and to permit manual rather than verbal responding. We predicted that, with this test, children would indeed learn and retain word forms after ostensive training and that they would perform similarly on the form, link, and link-generalization tests. Additionally, we were interested in exploring performance on the form test to determine whether there was evidence that failures were due to partial representations as evidenced by frequent selection of near-neighbor foils. To better understand the spatially supported form test itself, we explored children’s preferred response modality (pointing to the dot corresponding to the word form selection and/or producing the word form selection). We also related performance on the form test to an independent measure of phonological short-term memory. If we were successful in minimizing phonological memory demands with this task, then we should find no correlation. Finally, we were interested in accounting for any variability of performance on the form test within and across children. Thus, we examined performance in relation to word level factors (whether one or two syllables long) and learner factors (gender, age, and tendency to imitate) that have been reported to influence children’s recall of newly learned words (Baddeley et al., 1975; Huttenlocher et al., 1991; Weismer and Hesketh, 1996; Gathercole et al., 1997; Smith and Vela, 2001; Gathercole, 2007; Alt and Suddarth, 2012). Within subjects, we predicted that children would demonstrate better memory for shorter than longer words. As is common in the developmental literature, across subjects we predicted that variability would reflect better performance by girls than boys and by older than younger children (Fenson et al., 1993). Also, given past work which provides evidence that imitation is positively related to word learning (Bloom et al., 1974; Dore et al., 1976; Killen and Uzgiris, 1981; Masur, 1995, 2000; Masur and Eishorst, 2002) we predicted that children who imitate more often during training would remember more words during test than those who imitate less.

## MATERIALS AND METHODS

### PARTICIPANTS

The researchers obtained ethical approval for the current research through the University of Iowa’s Institutional Review Board (Approval Number = 00003007). Participants included sixteen 4- to 6-year-old children (mean age = 5.4, 64.75 months; males = 10, females = 6). The parents of the children completed from 12 to 23 years of education (mean = 17.05, SD = 3.41). The data from four additional participants was excluded: two due to experimenter error, one because she failed the hearing screening, and one because she did not attend the second session. One child was exposed to one word form 10 instead of 5 times during training. However, because her responses to this form were at chance during the tests, her data was retained.

### STIMULI

**FIGURE 2:**
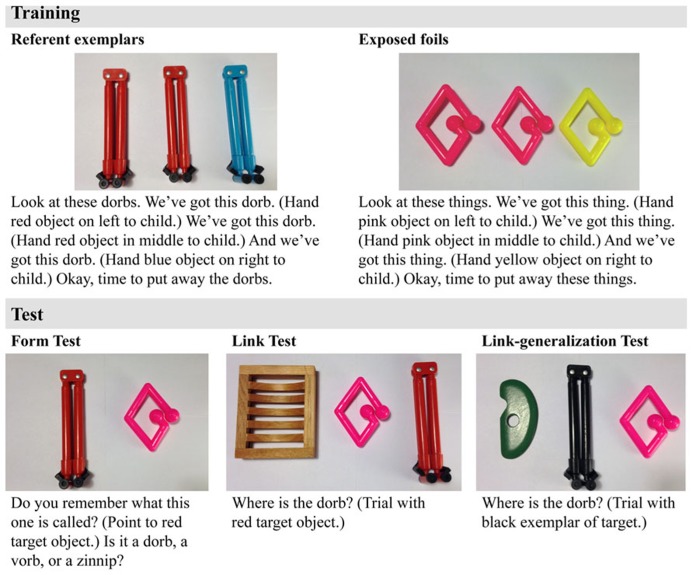
**Script used during the training and testing procedure**.

The objects presented to the children served as referent exemplars, exposed foils, unexposed foils, and generalization exemplars. Objects were excluded if, during pilot testing, several parents indicated that their children would have a label for the object or if several children easily labeled the object during training. Children were presented with multiple exemplars (in this case 3) of each target object during training. Two exemplars of each target object were identical to establish the category and the third varied from the other two in size, color, and/or shape to instigate generalization (Rost, 2011; see **Figure 2**). There were 12 referent categories.

Similar to the referent categories, each exposed foil category (12 total) was composed of two identical objects and a third varying from the other two in size, color, and/or shape. Twelve objects that children had not seen during training were used as unexposed foils during the link test and 12 additional unexposed foils were used during the link-generalization test. Twelve unexposed exemplars of the referent category (that differed from the trained exemplars in color, size, and/or shape) were used as the generalization referents during the link-generalization test. Objects presented on a given trial varied by only two properties (i.e., size, color, and/or shape). See **Figure 2** for examples.

Each referent category was randomly paired with one of twelve novel words (e.g., dorb), six of which were monosyllables and six of which were disyllables. To maximize learnability, all of the words had low lexical neighborhood densities and high phonotactic probabilities, and no two words shared the same initial syllable (Storkel and Adolf, 2009). The words were variable in terms of the place and manner features represented, but all were composed of early acquired sounds. Twenty-four novel words served as foils in a 3AFC form test. In a given trial of this test, one novel word (e.g., vorb) was a near neighbor that varied from the target form (e.g., dorb) in either onset, medial, or final consonant sound with the position of the change counterbalanced across target words; the other novel word (e.g., zinnip) differed from the target in number of syllables and majority of segments. Thus, two-syllable unrelated words were always used with one-syllable target words and vice versa. The target words were divided into two sets. Each set contained three monosyllabic and three disyllabic words and each set was similar in the place and manner features represented.

### PROCEDURE

All children passed a pure-tone audiometric screening administered in a non-soundproofed laboratory room at 0.5, 1, 2, and 4 kHz at 30 dB in at least one ear. Before training, the children completed a non-word repetition test [NWR (Dollaghan and Campbell, 1998)] to test their phonological working memory.

Training was divided into two parts, each involving the presentation and naming of six form-referent categories and the presentation (without naming) of six foil categories. The order of particular referent and foil presentations was randomized across the two parts of the training and across children. During training, the three exemplars of a given referent category were placed on top of the cloth and named five times (two times in the plural form, and three times in the singular form, see **Figure 2**). For example, the child was told, “Look at these dorbs. We’ve got this dorb, we’ve got this dorb, and we’ve got this dorb.” The child was encouraged to handle all of the objects after which the experimenter said, “Ok, time to put away the dorbs.” The presentation of a target referent category was followed by the presentation of a foil category. The foils were exposed during training to eliminate the possibility that during test, children would select targets based on familiarity alone. Part one continued until the first six referent and foil categories were presented. After a break of several minutes, part two of training continued in the same manner as part one.^1^

One week later, the child returned for the retention tests. Testing always began with the 3AFC form test designed to test the child’s ability to remember the phonological forms of each target word. This test was administered prior to the other tests because in the other tests the experimenter would state the correct labels for the objects. In this test, the child was presented with one target referent and one exposed foil (see **Figure 2**). The exposed foil was presented so that it would remain as salient as the target on subsequent tests; the child’s attention was drawn to the exposed foil, but she was not asked to name it (i.e., Do you remember that we looked at this one?). Then, the experimenter asked the child what the target referent was called and presented three word forms for the child to choose from: the target word, the near neighbor word, and the unrelated word (see stimuli section above). As the experimenter produced each word form, she pointed to one of three large black dots on a piece of paper. For example, the experimenter pointed to the target referent and asked the child, “Do you remember what this one is called?” Then, she pointed to the first black dot on a piece of paper and asked the child, “Was it a dorb?”; she pointed to the second black dot on the paper and asked the child, “Was it a vorb?”; and she pointed to the last black dot on the paper and asked the child, “Was it a zinnip?” (see **Figure 1**). The order of the target word, near neighbor and unrelated word was randomized across trials as was the order in which the experimenter asked about the exposed foil and the target referent. Either pointing to the representative dot or saying the correct word form was taken as a correct response. Children did not receive feedback about their responses on this or any of the tests so that responses to earlier tests would not influence responses to later tests. Each child received two practice/training trials with familiar objects (e.g., a car and a book) before they were asked about the target objects. Performance in the practice trials verified that children readily understood this test and had little difficulty either producing the word or pointing to the representative dot.

Next we administered the 3AFC-link test designed to test the child’s memory of the form-referent links. During this test, the child was shown one target object, one exposed foil, and one unexposed foil and was asked to find the target referent when the experimenter said its name (Where is the dorb? Can you point to the dorb?) Finally, the experimenter administered a 3AFC link-generalization test, designed to test the child’s ability to generalize the trained forms to a new exemplar of the trained referent. In this test, the child was shown a novel exemplar of the target referent, an exposed foil, and an unexposed foil and asked to find the target referent when the experimenter said its name. Different unexposed foils were used in the link and link-generalization tests to guarantee that they would be completely novel in both tests. However, the same exposed foils were used during the link- and link-generalization tests to guarantee that the foils were equivalently familiar. The objects’ (i.e., the target, exposed foil, and unexposed foil) relative location on the table was randomized across trials. Across training and all three tests, word-object pairs were trained and tested in the same order within participant. For example, if the child was presented with the “dorb” first during training, then they were tested with “dorb” first in the form, link, and link-generalization tests.

## RESULTS

**FIGURE 3:**
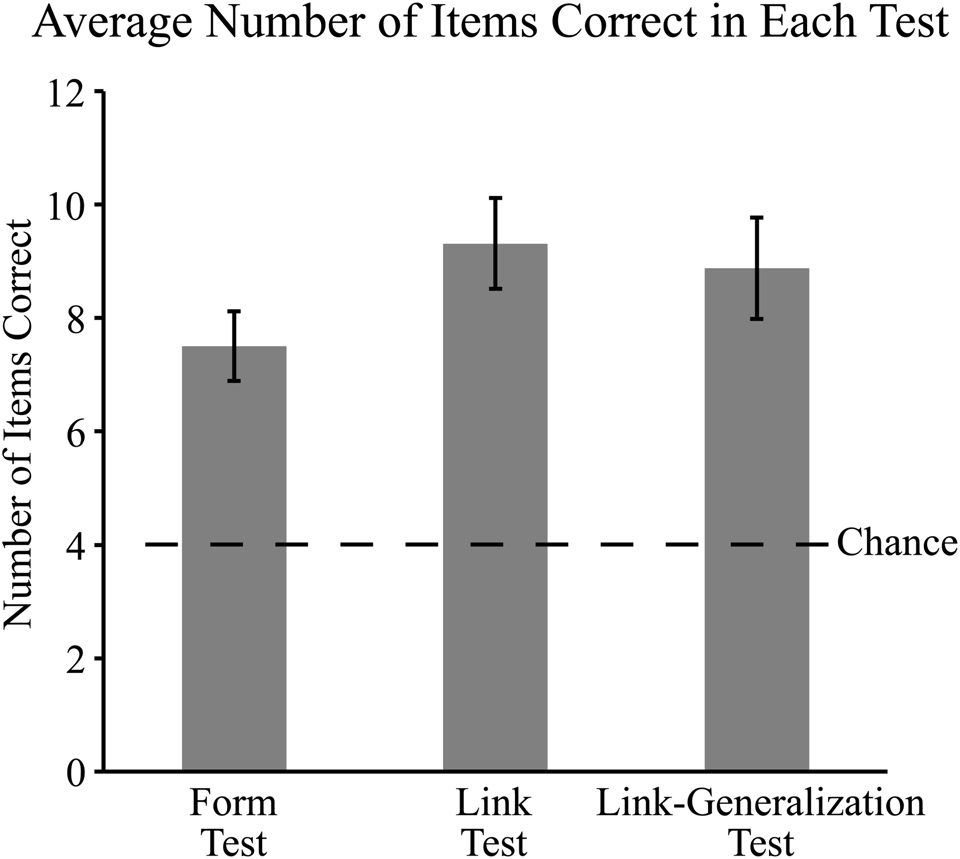
**Average number of items correct (out of 12) and standard errors in the form, link, and link-generalization tests.** Children’s responses on all three tests were significantly above chance and children’s responses on the form test were significantly lower than responses on the link test.

As predicted, children did retain trained word forms over the week-long interval (see **Figure 3**). Of the 12 words trained, the children recognized, on average 7.50 (SD = 2.45) forms, which was above chance, *t*(15) = 5.72, *p *< 0.001, *d *= 2.95. Children also remembered an average of 9.31 (SD = 3.20) of 12 word-referent links which was above chance, *t*(15) = 6.64, *p *< 0.001, Cohen’s *d *= 3.43, and 8.88 (SD = 3.59) out of 12 word-referent links on the link-generalization test which was also above chance, *t*(15) = 5.43, *p *< 0.001, Cohen’s *d *= 2.80. We compared children’s performance on the three tests through a repeated measures ANOVA with number of correct responses on each of the three tests as the dependent variable. This revealed a significant effect of test type, *F*(2,30) = 5.23, *p* = 0.01, $\eta_{p}^{2}$ = 0.26. Although we predicted that children would perform similarly in the link and form tests given the reduced task demands of the form test, a Bonferroni *post hoc* test revealed that children were correct significantly more often on the link test than the form test, *p* = 0.01. Responses to the link generalization test did not differ significantly from the form test, *p* = 0.08, or the link test, *p* = 1. Scores on the three tests were significantly correlated with each other: form and link, *r* = 0.72, *p *= 0.002; form and link-generalization, *r* = 0.70, *p *= 0.003; and link and link-generalization, *r* = 0.80, *p* < 0.001.

As one of the purposes of the current study was to investigate the usefulness of an AFC test of word form, further analyses were conducted to explore children’s responses in the form test. First we examined the influence of the near neighbor foil (**Table 1**). We ran a repeated measures ANOVA by item (target, near neighbor, or unrelated) with number of times participants selected each response as the dependent variable. The result was a significant effect for response type, *F*(2,30) = 25.98, *p* < 0.001, $\eta_{p}^{2}$ = 0.63. A Bonferroni *post hoc* test revealed that targets were selected more often than near neighbor foils, *p* = 0.001, and unrelated foils, *p* = 0.001. Selections of near neighbor and unrelated foils did not differ, *p* = 1.

**Table 1:** Means, standard deviations (in parentheses), and range of children’s response to each option in the form test.

Form Test		
Target word	Near neighbor foil	Maximally different foil
Mean = 7.50 (2.45)	Mean = 2.38 (1.50)	Mean = 2.19 (1.72)
Range 2–11	Range 0–6	Range 0–5

We also asked whether the type of near neighbor presented during each trial influenced the likelihood that children would select the near neighbor. Of the near neighbors in the form test, three differed from the target word in initial consonant, four differed in the medial consonant, and five differed in final consonant. The number of times that children selected the near neighbor for each type of variation (initial, medial, or final) were calculated, and converted to a percentage to compare them. A repeated measures ANOVA by subjects with percentage of near neighbor foil selections per position as the dependent variable revealed no effect, *F*(2,30) = 0.43, *p = *0*.*66, $\eta_{p}^{2}$ = 0.03.

Next we examined the children’s response behaviors. From video recordings of the tests, we coded whether children responded verbally, pointed to a dot, or did both in their response (regardless of whether their response was correct). For three children, the audio track was too noisy to hear to code accurately. For the 13 remaining children, we analyzed how many of the 156 forms (12 forms each for 13 children) included each type of response. Children just pointed for 71 or 46% of the forms (mean = 5.46 forms per child, SD = 5.61), just responded verbally for 46 or 29% of the forms (mean = 3.53 forms per child, SD = 4.70), and both pointed and responded verbally for 39 or 27% of the forms (mean = 3.00 forms per child, SD = 4.46). Overall children pointed for a total of 110 or 71% of the forms, and responded verbally for a total of 85 or 54% of the forms.

We also investigated individual differences in children’s response preferences. We found that the type of response that each child provided was fairly consistent across forms. For example, seven children pointed for all 12 forms (five of those children only pointed for all forms while two of those children both pointed and provided a verbal response for 7 and 8 forms, respectively), and five children provided a verbal response for all 12 forms (they both responded verbally and pointed for 7, 3, 2, 2, and 1 form, respectively). The remaining child only pointed for 2 forms, only responded verbally for 1 form, and both pointed and responded verbally for 9 forms.

When children both pointed and responded verbally, the two responses were consistent in 33 of 36 cases. Only three children pointed to a dot that was different from the form they stated (for example, saying “dorb” while pointing to the dot for “vorb”), and each of these three children only made this mistake once. In these cases, the child’s verbal response took precedent over their manual response when coding their answer. For children who produced a form (with or without pointing), they overwhelmingly correctly produced one of the forms presented to them (whether it was the target, near neighbor or distracter). Only three children mispronounced the forms: two children mispronounced 1 form each, and one child mispronounced 2 forms. In these cases, children’s verbal responses were coded as the form (either the target, near neighbor, or distracter) that was most phonologically similar to the form they produced. For only one mispronounced form did the child also point to a dot, and in this case the form pronounced was most phonologically similar to the form represented by the dot that they pointed to.

While most children waited to hear the three word-form options given before indicating their response, four of the children did provide alternate labels before the options were presented. In all cases when this occurred, children produced a form that was not phonologically similar to the target form (e.g., “a spinner” for the grod), suggesting that children were trying to guess what the object was called. These four children provided an alternate name for 6, 5, 2, and 1 of the forms, respectively. In all cases when this occurred, children selected one of the options once they were presented. Of the 14 forms this occurred with, children only identified the target word in three cases. This suggests that they were guessing once they heard the three options, and were providing an alternate form because they did not remember the target form.

A set of analyses addressed the extent to which the phonological working memory demands of the form test were sufficiently reduced. Data from the NWR test was collected for 12 children (two children were not administered the test due to experimenter error, and for two children, the audio recording of their responses was too difficult to hear in order to code). Correlations were calculated between children’s performance in the NWR test and performance in the word form, referent, and link-generalization tests. This analysis revealed that performance in the NWR tests was not significantly correlated with performance in the form test, *r* = 0.08, *p* = 0.81; the link test, *r* = 0.01, *p* = 0.97; or the link-generalization test, *r *= 0.21, *p* = 0.51. Theses results suggest that the form test, like the referent and generalization tests is relatively unaffected by phonological memory demands.

To explore whether children’s phonological working memory skills were related to the type of response they gave, correlations were conducted with children’s NWR score and whether they pointed, responded verbally, or did both in the form test. Of the 13 children with good quality video recordings, four children were excluded from the following analyses because they did not have a score for the NWR test. For the remaining nine children, the score on the NWR test was not significantly related to the number of times children pointed *r* = 0.20, *p* = 0.61, responded verbally *r* = -0.11, *p* = 0.78, or both pointed and responded verbally *r* = -0.19, *p* = 0.62.****

### LEARNER FACTORS

Although individual differences in accurate recognition of word forms were not related to differences in phonological working memory, there was ample variation to explain. The range of scores was 2–11 out of a possible range of 0–12. To explore whether this variation was related to sex, we ran a series of independent samples *t*-tests, but these revealed no effects of sex on children’s performance in the form *t*(14) = 0.20, *p* = 0.84, Cohen’s *d* = 0.11; link, *t*(14) = 0.18, *p* = 0.86, Cohen’s *d* = 0.09; or link-generalization test, *t*(14) = 0.82, *p* = 0.43, Cohen’s *d* = 0.45. To determine whether age was related to performance in each test, correlations were calculated. These analyses revealed no significant correlations between age in months and the number of target words selected, *r* = 0.33, *p *= 0.22; number of near neighbors selected, *r* = -0.35, *p *= 0.19; or number of maximally different foils selected, *r* = -0.23, *p *= 0.38 on the form test. Additionally, there was not a significant relationship between age and correct responses on the link, *r* = 0.24, *p *= 0.37, or link-generalization test *r* = 0.35, *p *= 0.18. Furthermore, to investigate whether the likelihood that child would point in the form test was related to their age, correlations were calculated between age and the number of times children just pointed, just verbally responded, or both pointed and verbally responded to indicate their choice of word form. Children’s ages were not significantly related to the number of times children pointed *r* = 0.07, *p* = 0.80, responded verbally *r* = -0.35, *p* = 0.18, or both pointed and responded verbally *r* = 0.36, *p* = 0.17 in the form test.

Occasionally children imitated the target word forms that the examiner produced during training. Correlations between the number of word forms imitated and the child’s performance revealed a significant negative relationship for the form, *r* = -0.57, *p *= 0.02; link, *r* = -0.76, *p *= 0.001; and link-generalization, *r* = -0.57, *p *= 0.02 tests. That is, the more children imitated, the poorer their learning outcomes.

### WORD-LEVEL FACTORS

Finally we examined the words themselves as a source of variation. Recall that neighborhood characteristics were comparable across words, but the words did vary in length. The number of one-syllable vs. two-syllable words that children recalled was compared through a paired samples *t*-test. Means suggest that children remembered more two-syllable (mean = 4.31, SD = 1.14) than one-syllable (mean = 3.19, SD = 1.80) words; however, this difference did not reach significance, *t*(15) = 1.86, *p* = 0.08, Cohen’s *d* = 0.74. Additionally, the experimenters noticed that, during the tests, children had a tendency to correctly identify more word forms from the referent-form set that was presented first during training (Mean = 3.38, SD = 1.54) than the referent-form set that was presented second during training (Mean = 1.06, SD = 0.85). To investigate whether set (A, B) and order (first set presented, second set presented) affected children’s responses, a series of repeated measures ANOVAs by item were conducted. The analysis of children’s responses in the form test revealed a significant main effect for order *F*(1,10) = 16.19, *p* = 0.002, $\eta_{p}^{2}$ = 0.62, but there was not a significant main effect for set *F*(1,10) = 0.15, *p* = 0.71, $\eta_{p}^{2}$ = 0.02, or a significant set by order interaction *F*(1,10) = 1.27, *p *= 0.29, $\eta_{p}^{2}$ = 0.11. The analysis for the link test revealed a significant main effect for order *F*(1,10) = 17.58, *p* = 0.002, $\eta_{p}^{2}$ = 0.64 and set *F*(1,10) = 6.32, *p* = 0.03, $\eta_{p}^{2}$ = 0.39 qualified by a significant order by set interaction *F*(1,10) = 6.17, *p* = 0.03, $\eta_{p}^{2}$ = 0.38. *Post hoc* analyses revealed that children scored lower in set B when it was presented second than when it was presented first. Furthermore, children scored lower in set B when it was presented second than set A when it was presented first. The analysis for the link-generalization test revealed a significant main effect for order *F*(1,10) = 59.55, *p* < 0.001, $\eta_{p}^{2}$ = 0.86 and set *F*(1,10) = 7.22, *p* = 0.02, $\eta_{p}^{2}$ = 0.42, again qualified by a significant order by set interaction *F*(1,10) = 59.55, *p* < 0.001, $\eta_{p}^{2}$ = 0.86. *Post hoc* analyses revealed that children scored lower in set B when it was presented second than all other presentations.

## DISCUSSION

We explored two primary questions in the current study. First, we investigated whether children would demonstrate long-term memory for word forms if they were asked to recognize the forms in a spatially supported test that allowed manual responding. Second, we investigated whether children’s long-term memory for word forms is comparable to their long-term memory of word-referent links when test demands are made to be more similar. We also determined whether the test itself was as supportive as we intended by verifying that the children indeed took advantage of the opportunity to respond manually, and by assessing whether phonological memory demands were sufficiently reduced. Finally, we explored word-level factors and learner factors to discern potential sources of variability in the children’s word-form memory.

Critically, the spatially supported form recognition method used in the current study was sensitive to children’s long-term memory for word forms. After training, which involved ostensive naming of 12 novel words at a rate of five times per word, the 4- to 6-year-old participants correctly recognized an average of 7.5 words after a one-week retention interval. Thus, children in the current study showed better performance when tested via recognition than the verbal recall performance reported in previous training studies that also involved ostensive naming and a sizable retention interval. For example, the 8-year-old participants in McGregor et al. (2007) recalled only 4.29 of 20 words and the 2- to 3-year-old participants in Munro et al. (2012) recalled only 0.33 of eight words. The method presented here allowed the children to demonstrate their memory for word forms before they were likely to recall and produce these forms. Again, it is important to point out that we are not suggesting that recognition tests should replace verbal recall tests as these tests are measuring different abilities in children. Instead, we posit that the spatially supported form recognition test is a tool that researchers and clinicians can use to assess early stages of the child’s developing memory for word forms.

Several key aspects of the recognition test supported the children in demonstrating their memory for word forms. Motor demands were reduced in that children were allowed to indicate their choice manually instead of verbally. The children indicated their response via pointing alone on 46% of all form recognition test questions. Thus, if children had been tested using traditional verbal recall methods, they might have provided no response in almost half of the questions asked of them. In the vast majority of pointing plus verbal responses, children pointed to the dot associated with the word form they produced, suggesting that children have little difficulty associating a visual-spatial cue with the word forms despite the fact that forms had no iconic associations with the dots. Moreover, phonological working memory demands were reduced by use of the three dots to ground the fleeting word stimuli in space. Evidence for this is the lack of correlation between the children’s working memory abilities as measured by the NWR test and their form recognition outcomes. Through future research we can explore whether this method also effectively reduces the working memory demands on children with particularly poor phonological working memory skills (e.g., children with LI, or younger children) who would have a hard time remembering the three alternative forms presented by the researcher without the additional visual-spatial cue.

Despite strong performance on the word form recognition test, none of the children demonstrated memory for all of the trained word forms. For the word-form test, the most powerful predictor of learnability was order of training, an effect that we had not anticipated. The children remembered more items from the first six-item training set than the second. Horst and Samuelson (2008) found a similar effect in that 24 month-olds were better able to remember the first four word-referent links they were exposed to during training than the last four word-referent links. While the current study does not address the question of whether this is a limitation of encoding, consolidation, or retrieval, past research suggests that encoding tends to be a bottleneck for young children’s word learning (Munro et al., 2012). This, together with the fact that memory for the first trained set was stronger than the second motivate the hypothesis that proactive interference of encoding limits the number of words that one can learn in a given period.

There was also a wide range of performance; one child recognized only 2 of the trained word forms but another recognized 11. Neither sex nor age accounted for this variability among the children. We predicted that children who practiced during training by imitating the examiner might be at an advantage but, to our surprise, the opposite was true. The more imitative children performed more poorly than the less imitative children on all three tests: form, link, and link-generalization. This stands in contrast to previous reports of a positive relationship between imitation of novel words and later word learning (Bloom et al., 1974; Dore et al., 1976; Killen and Uzgiris, 1981; Masur, 1995, 2000; Masur and Eishorst, 2002). Interestingly, Leonard et al. (1983) found that imitation was positively related to infants’ later production of word forms, but was not related to their comprehension of the forms as measured in a typical form-referent link recognition task. Unfortunately it is difficult to reconcile these inconsistencies as the previous studies included children who were considerably younger than the children in the current study. Imitation could play a different role for learners of different ages or abilities. Perhaps preschool-age children who are better able to encode novel word forms may not feel the need to imitate the forms during training, whereas children who have difficulty remembering the forms may repeat them in an attempt to better remember them. This too is a fruitful line for future research.

The second main question we explored was whether children would perform as well on tests of word forms as tests of word-referent links once test demands were made to be more similar. This was not the case. Children still performed significantly better in the link test than the form test. The sequential and fleeting nature of word forms likely represent a more difficult target and one that is perhaps served by different memory systems than the pairing of that word to its referent (see Martin and Gupta, 2004) As a result, word forms may require a more extensive level of training (e.g., more exposures, more training sessions) or training in smaller sets (e.g., not more than six per session) to reach comparable levels of recognition of word-referent links.

Overall, the current study provides evidence that children do learn word forms after brief ostensive naming exposures and that spatially supported AFC recognition is a sensitive measure of children’s memory for word forms. It is our hope that through the implementation of this measure, researchers will gain a better understanding of children’s capacity to encode and retain word forms especially during early stages of word learning. It is of particular interest to us to determine whether these methods will provide a useful tool for speech-language clinicians and researchers to assess word form learning among children who struggle in traditional verbal recall tests (such as younger children and children with low phonological working memory skills). Through the application of these methods in the future, researchers can better uncover points of break-down in word learning as well as word-level and individual factors that predict children’s ability to learn word forms.

## AUTHOR CONTRIBUTIONS

Dr. McGregor conceptualized the recognition method of assessing memory for word forms. Both Dr. McGregor and Dr. Gordon designed the research methods, and conducted the data analyses. Dr. Gordon collected and coded all data, and wrote the current manuscript with significant feedback from Dr. McGregor.

## Conflict of Interest Statement

The authors declare that the research was conducted in the absence of any commercial or financial relationships that could be construed as a potential conflict of interest.

## Footnotes

1 The data analyzed here were collected as part of a larger, unpublished study on the role of physical context in memory for new words. Specifically, to explore whether training and testing in the same room facilitated memory more than training and testing in different rooms, the children were exposed to the first referent and foil set in room A (or B) and the second in room B (or A) and they were tested for both sets in a single room (either A or B). However, a preliminary analysis revealed no differences between testing in a matching vs. a non-matching context for the form test, *t*(15) = 1.16, *p* = 0.26, Cohen’s *d* = 0.38; the link test *t*(15) = 0.35, *p* = 0.73, Cohen’s *d* = 0.10; or the link-generalization test *t*(15) = 1.28, *p* = 0.22 Cohen’s *d* = 0.12. Moreover, because these conditions were within-subject and order of sets and assignment of rooms was randomized among children, this manipulation is irrelevant to the interpretation of the current results.
